# Leveraging Administrative Data Linkages to Study Waterborne Lead Exposure in Longitudinal Aging Cohort Studies

**DOI:** 10.1093/geroni/igaf122.1460

**Published:** 2025-12-31

**Authors:** Victoria Marino, Joseph Ferrie, Mina Antic, Ashley Dorame, Avron Spiro, Robert Waldinger, Daniel Mroczek, Lewina Lee

**Affiliations:** VA Boston Health Care System, Boston, Massachusetts, United States; Northwestern University, Evanston, Illinois, United States; Massachusetts General Hospital, Boston, Massachusetts, United States; Boston University - Chobanian & Avedisian School of Medicine, Boston, Massachusetts, United States; Boston University - Chobanian & Avedisian School of Medicine, Waban, United States; Harvard University, Cambridge, Massachusetts, United States; Northwestern University, Evanston, Illinois, United States; VA National Center for PTSD & Boston University Chobanian & Avedisian School of Medicine, Boston, Massachusetts, United States

## Abstract

Lead (Pb) is a neurotoxin with lasting cognitive, behavioral, and physiological effects, particularly when exposure occurs in early childhood. Nearly half of the U.S. population today experienced Pb exposure during this critical developmental period, yet few studies have examined its long-term health consequences using a life course perspective. We aim to demonstrate the feasibility of aging cohort studies to construct a phenotype of early childhood Pb exposure via administrative data linkages. The Boston Early Adversity and Mortality Study integrated municipal, state, and federal records with data from three Boston-based, socioeconomically diverse longitudinal cohorts (the Normative Aging Study [NAS] and the Grant and Glueck [GG] cohorts of the Harvard Study of Adult Development) and added siblings (NBEAMS=13,151; nNAS=9,972, nGG=3,179). Pb exposure was operationalized using linkage-derived information on water service line materials (non-Pb, Pb, mixed Pb and non-Pb) and municipal water pH (continuous) anchored to participants’ residential home address at birth. We examined Pb exposure patterns by childhood SES status. We identified birth locations for 99% of the 12,559 U.S.-born BEAMS participants, spanning 688 unique city-state combinations. Pb exposure could be estimated for 52% of 688 birth locations, covering 93% (n = 11,654) of U.S.-born BEAMS participants. Pb exposure followed a socioeconomic gradient, χ2(4)=1,035.9, p<.0001, such that 90%, 66%, and 37% of the lowest- (Glueck), middle- (NAS), and highest- (Grant) SES cohorts, respectively, had the highest Pb exposure levels. We discuss the value of administrative data linkages in reconstructing environmental exposures and advancing life course research.

